# User-centered virtual environment design for virtual rehabilitation

**DOI:** 10.1186/1743-0003-7-11

**Published:** 2010-02-19

**Authors:** Cali M Fidopiastis, Albert A Rizzo, Jannick P Rolland

**Affiliations:** 1 School of Health Professions, University of Alabama-Birmingham, Birmingham, AL, USA; 2 Integrated Media Systems Center, University of Southern California, Los Angeles, California, USA; 3 Institute of Optics, University of Rochester, Rochester, NY, USA

## Abstract

**Background:**

As physical and cognitive rehabilitation protocols utilizing virtual environments transition from single applications to comprehensive rehabilitation programs there is a need for a new design cycle methodology. Current human-computer interaction designs focus on usability without benchmarking technology within a user-in-the-loop design cycle. The field of virtual rehabilitation is unique in that determining the efficacy of this genre of computer-aided therapies requires prior knowledge of technology issues that may confound patient outcome measures. Benchmarking the technology (e.g., displays or data gloves) using healthy controls may provide a means of characterizing the "normal" performance range of the virtual rehabilitation system. This standard not only allows therapists to select appropriate technology for use with their patient populations, it also allows them to account for technology limitations when assessing treatment efficacy.

**Methods:**

An overview of the proposed user-centered design cycle is given. Comparisons of two optical see-through head-worn displays provide an example of benchmarking techniques. Benchmarks were obtained using a novel vision test capable of measuring a user's stereoacuity while wearing different types of head-worn displays. Results from healthy participants who performed both virtual and real-world versions of the stereoacuity test are discussed with respect to virtual rehabilitation design.

**Results:**

The user-centered design cycle argues for benchmarking to precede virtual environment construction, especially for therapeutic applications. Results from real-world testing illustrate the general limitations in stereoacuity attained when viewing content using a head-worn display. Further, the stereoacuity vision benchmark test highlights differences in user performance when utilizing a similar style of head-worn display. These results support the need for including benchmarks as a means of better understanding user outcomes, especially for patient populations.

**Conclusions:**

The stereoacuity testing confirms that without benchmarking in the design cycle poor user performance could be misconstrued as resulting from the participant's injury state. Thus, a user-centered design cycle that includes benchmarking for the different sensory modalities is recommended for accurate interpretation of the efficacy of the virtual environment based rehabilitation programs.

## Background

Over the past 10 years, researchers have explored the use of virtual environments (VEs) as a rehabilitation tool [1-4]. Although studies have documented successful re-training and transfer of training while utilizing this paradigm [5,6], there are few studies that suggest methods of designing VEs that transition from specific applications of cognitive re-training to comprehensive rehabilitation training programs [7,8]. Given that most VE applications for cognitive retraining require customized applications [9], cost effectiveness is an initial design consideration [7]. However, there is some evidence that when designed following a user-centered design cycle, VE platforms can be validly and reliably applied across therapy scenarios [10].

"Good Fit" assessments are another suggested requirement of the virtual rehabilitation (VR) design cycle. The purpose of these assessments is to gauge how well the VE solution presents real world attributes in a more controlled, repeatable manner that will allow for comparable results over treatment effects [10]. This point raises an important issue: VE solutions for cognitive rehabilitation are mostly designed to capture data necessary to evaluate levels of cognitive function or transfer effects pre and post rehabilitation. As such, they are inherently guided by experimental design and scientific principles. This fact argues for standardized design methodologies when constructing VR environments, especially for applications that target persons with cognitive impairments. Lack of standardization leads to redundancy of VE applications and platforms; more importantly, it makes comparisons across research endeavors difficult [11].

International guidelines do exist for designing computer-based systems that are user-centered and iterative throughout the design lifecycle. Specifically, the International Standard ISO13407, the Human-Centered Design Process for Iterative Systems, outlines principles of human-centered design that account for user context, computer-system design, and environment of use within an iterative design cycle [12]. Usability evaluation, ease of use and utility, is a key component to the user-centered design methodology. The main goals of usability within the design cycle are to ensure system effectiveness, efficiency, safety, and utility [13]. VE based trainers for medical and military applications involving person without cognitive impairments have been successfully designed using the ISO 13407 framework [14,15]. However, satisfying the recommended guidelines is a subjective endeavor and determining valid usability testing for persons with impairments such as anterograde amnesia may require more medical community agreement.

Further, Stanney [16] contended that human sensory and motor physiology in general may prove to be limiting factors in some aspects of VE design. The Human-Computer Interaction (HCI) community has proposed varying general VE systems design approaches including those that focus on perceptual issues [17,18], usability [19,20], or combined perceptual and usability models [21,22]. Yet, there are several technological and computer graphics issues that lead to degraded perception in VEs that may confound VE rehabilitation assessments [23].

For example, a VE system may utilize a head-worn display (HWD). Microdisplays within HWDs typically limit the user's visual resolution acuity [23]. Further, HWD optical systems with a single imaging plane may also affect the natural accommodation and convergence mechanisms of the human visual system, thereby degrading depth cue information [24]. The resulting visual performance errors have the potential to distort experimental results, including those obtained from brain imaging.

**Figure 1:**
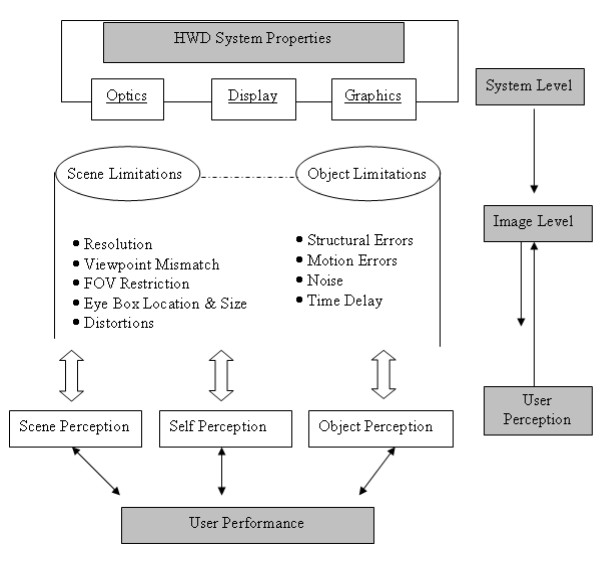
**User-centered VE design approach example**. Modified user-centered approach to the head-mounted display design cycle adapted from R.G. Eggelston (1997).

When evaluating VE system design, separating the human component from the engineering component may prove difficult [25]. Melzer and Moffit [26] addressed this issue by applying user-centered methodologies to HWD design cycles. Figure 1 represents an example of a user performance model for HWDs adapted from Eggleston [27]. The user performance model outlines the interdependencies of HWD properties, computer graphics techniques, and their combined effects upon the user's perception of the VE. More specifically, the model illustrates how errors in the hardware (HWD optics and display) and software (Computer Graphics) impact the user's ability to correctly perceive the constructed VE space. The user also contributes his or her individual differences in perceptual abilities (e.g., spatial processing) to the overall error. Thus, the model must also include a user perception to image level two-way interaction as illustrated in Figure 1.

Following the user-centered design model, the HWD designer is not only responsible for usability from a user's perspective, but from the software perspective as well. Thus, HWD designers are concerned with limits in HWD parameters such as display resolution (image quality), field of view (information quantity), and contrast (light intensity changes) [28]. Just as with user-centered HWD design, user-centered VE design considers the standard limits of the human visual system (e.g., visual acuity, contrast modulation, and stereoacuity) as minimal user requirements for optimal viewing of the VE scene [29]. In contrast, some researchers suggest that visual errors may be caused more by the graphical techniques used to define the spatial layout of the VE [30,31]. Thus, even with a well designed and calibrated HWD, the VE may not support proper viewing conditions for successful task completion.

**Figure 2:**
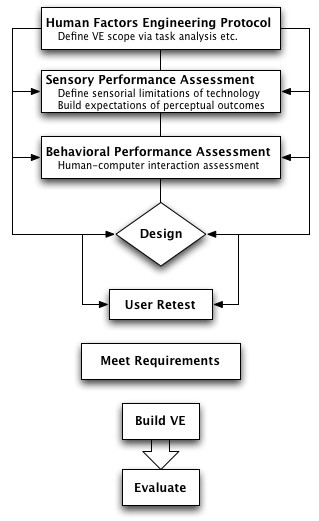
**Proposed iterative VE design cycle**. Proposed interactive iterative VE design cycle including sensory performance metrics for establishing baselines within a cognitive rehabilitation VE.

To circumvent these technology, graphics, and user issues, an interactive and iterative VE design cycle that includes sensory performance metrics for establishing baselines within a cognitive rehabilitation VE is proposed and diagramed in Figure 2. The design cycle integrates the requirements of the International Standard ISO13407, while including components of the more successful HCI VE design guidelines [22]. Sensory performance is measured before and during the design phase to ensure that the technology assembled is appropriate for the rehabilitation protocol. In addition, performance baseline metrics are obtainable. These metrics allow for the cross comparison of VE rehabilitation systems and the means to separate user performance from technology limitations during experimental analysis.

The VE rehabilitation system is not built until the component technology and graphical methods meet the task requirements for the rehabilitation protocol. A more important outcome of this methodology is that rehabilitation specialists can understand empirically the best VE system designs for providing effective treatment for persons experiencing cognitive impairments. Thus, the VE rehabilitation application is extendable to a successful, cost effective, and comprehensive rehabilitation program.

An ongoing impediment to VE system design is that usability assessments lack appropriate sensory tests (vision, auditory, smell, and touch) to provide accurate benchmarks for VE systems (i.e., technologies, computer graphics, and users). As a step toward narrowing this gap, we present modules of a vision test battery that quantifies key components of the human image processing system, namely resolution visual acuity and depth perception modules [23,32,33]. In this paper, we shall present results obtained with the stereoacuity module. The test battery can be performed when considering different types of VE methods (e.g., augmented reality) as well as with varying types of display technologies (e.g., projectors, monitors or HWDs). The results of such a battery should provide basic and applied vision parameters for the total VE system, which will allow for appropriate benchmarks and performance evaluations that control for visual errors (e.g., distorted depth) within VE based cognitive rehabilitation applications.

## Methods

### HWD technology

The purpose for performing tasks while wearing a see-through HWD is that real and virtual world objects are combined to make up the task space. For example, some types of therapy may be best performed utilizing virtual components (e.g., stove burners) along with real world objects (e.g., dials for setting heat) instead of replicating the total rehabilitation setting in a solely virtual counter part. There are two choices for see-though displays and they are categorized based upon how they merge the real and virtual scene: optical see-through HWDs employ a semi-transparent mirror, while video-see through HWDs use a video camera (see [34] for a comprehensive review).

**Figure 3:**
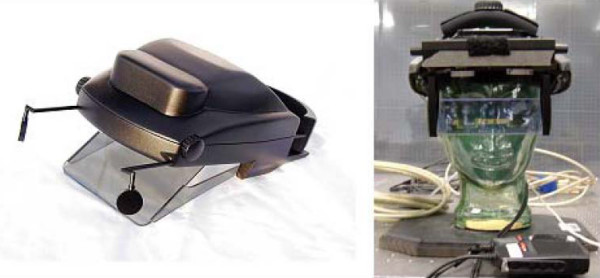
**Optical See-through HWD prototypes**. First (left) and second (right) generation prototype head-mounted projection displays developed in the University of Central Florida.

Optical see-through HWDs are typically associated with augmented reality tasks whereby the virtual world is overlaid onto real objects [35]. Figure 3 pictures two optical see-through displays, first and second generation prototype head-worn projection displays (HWPDs) whose optics were developed in the ODALab at the College of Optics and Photonics at the University of Central Florida [36]. Because the original stereoacuity assessment was conducted using the bench prototype of the first generation HWPD (HWPD-1), the HWPD-1 and HWPD-2 (second generation) were used in the experiment.

### HWPD parameters

Table 1 Technology specifications for HWPD-1 and HWPD-2 used in experiments. Table 1 provides the technical specifications for the HWDs worn during the stereoacuity testing. Information such as display type, field-of-view (FOV), interpupillary eye distance (IPD) range, and resolution are important parameters of the HWD that determine the users' visual performance. For example, resolution as imposed by the microdisplay can be estimated by measuring the average subtense of a single pixel in either the horizontal or vertical dimension after being magnified by the optics. This resolution value can be computed from the horizontal or vertical resolution given in pixels and the FOV for that dimension. The approximated resolution can then be compared to that of the human visual system. The resolution visual acuity of the human eye is accepted as 1 arc minute or 20/20 [37]. Comparatively, the measured resolution visual acuity for users wearing the HWPD-1 is 4.1 arc minutes (~20/80) and 2.73 arc minutes (~20/60) for the HWPD-2 [38]. Thus, the user wearing the HWPD-2 should be able to see better object detail than the person wearing the HWPD-1; however, factors such as display brightness as determined by the display type (e.g., liquid crystal or organic light-emitting) and graphical content can play a role in detail visibility.

**Table 1:** HWD technology specifications. HWPD-1 and HWPD-2 specifications used in the experiments.

		FOV (Degree)		Resolution (Pixels)				
**HMD Type**	**Display Type**	**H**	**V**	**H**	**V**	**Display Size** **mm**	**Focus Plane** **mm**	**IPD mm**

HMPD-1	LCD	41	31	640	480	27 × 20	Infinity	55-75
800		40	31				800	
1500		41	31				1500	
3000		42	32				3000	

HMPD-2	OLED							
800		33	25	800	600	12 × 9 mm	800	55-72
1500		34	26				1500	
3000		34	26				3000	

Canon	LCD	57	37	640	480		2000	63

VR6	LCD	48	36	640	480	1.3 × 2.59	914	52-74

a Horizontal and Vertical

b Depth of the optical plane (i.e., depth at which the image is rendered)

Most often, researchers do not report the optical depth plane of the HWD; however, this parameter is critical to understanding visual perception issues in VEs. The virtual image created by the HWD is usually magnified and presented at a fixed distance from the observer, usually between 500 mm and infinity [39,40]. This fixed distance is based upon the optics of the HWD system and may result in conflicts between the accommodation and the convergence mechanisms of the eye. Although multi-focal plane HWDs have been proposed [41], they are not available on today's HWD market. As a result, the focus distance of HWDs does not dynamically change as does the human eye to allow for focus on near or far objects. Because both optical see-through displays were custom built, we could adjust the focus planes for both HWPDs to present the virtual image at different viewing depths from the observer. The importance of this adjustment is that the virtual image and the rendered image are collocated on the optical plane, thus eliminating the mismatch between accommodation and convergence mechanisms of the observers' eyes.

The optics for HWPD-1 was optimized for infinity viewing (i.e., viewing distances > 6 m) by design, thus the optical depth plane for this display is typically set to display images at infinity. However, the optics of this display also allow for adjustments to the optical depth plane, and thus allow for viewing distances of 800, 1500, or 3000 mm with only a slight decrement in resolution. Comparatively, the optics for HWPD-2 were optimized to technically operate at viewing depths of 800, 1500, and 3000 mm Because of this inherent design specification, the adjustments of the optical depth plane for HWPD-2 do not imply a compromise in image resolution. In the forthcoming experiments, we assessed the participants' stereoacuity at 800, 1500, and 3000 mm to confirm the depth presentation capabilities of each display.

It is important to note that the mismatch between the accommodation and the convergence mechanisms of the human eye is also accentuated by computer graphics techniques. More specifically, computer graphics render objects under infinity viewing conditions because the virtual cameras are considered as single fixed points or eyepoints [42]. How computer graphics techniques interact with technology constraints to impact user performance is another reason that establishing perceptual baselines are important to include in studies that involve learning or retraining.

### Stereoacuity benchmark test design

Wann and Mon-Williams [17] argued that VEs should support "salient perceptual criteria" such as binocular vision that allow for the appropriate perception of spatial layout, which in turn supports naturalistic interaction (p. 835). Their contention that VEs design must center upon the perceptual-motor capabilities of the user is an important design criteria for extending VEs to rehabilitation scenarios. Rehabilitation scenarios involving Activities of Daily Living (ADLs) may necessitate a level of complexity and realism beyond simple reaching tasks and manipulating virtual objects to traversing a virtual grocery store and handling real objects. In addition, correct spatial locations of objects within a virtual space may be necessary to support transfer of training to the home.

Visual performance testing may be difficult since when viewing a mixed reality scene the visual abilities of the user are dependent upon the sensory characteristics of the virtual and real objects (e.g., brightness and contrast) as well as the layout of the VE space [43]. Further, the human eye is an optical system that is functionally limited much like the HWD in such parameters as resolution. Given that the HWD parameters are designed with respect to limitations of the human eye, clinical vision tests which elucidate the functional limitations of the human eye are applicable to testing visual performance when the human eye is coupled with an HWD. Thus, we chose vision tests associated with established methodologies and real world correlates.

Clinical tests for stereoacuity can be divided into two categories, real-depth tests and projected-depth tests. The Howard-Dolman two-peg test is a classic example of a real-depth test whereby a real test object is moved in and out of the plane of one or more target objects [44]. The amount of difference in alignment between the two objects determines stereoacuity sensitivity. Stereograms, which present left and right eye perspective views of an image to the viewer, are examples of projected-depth tests. Although projected-depth tests are capable of eliminating most secondary depth cues, which diminish the accuracy of real-depth tests, the presentation of these tests are not reliable in VEs [45]. The modified virtual Howard-Dolman task (V-HD task) developed by [32] and later improved upon by [33] qualifies in general as a projected-depth test. At this time, the assessment provides the best metric for measuring stereoacuity with regard to VE system assessment.

**Figure 4:**
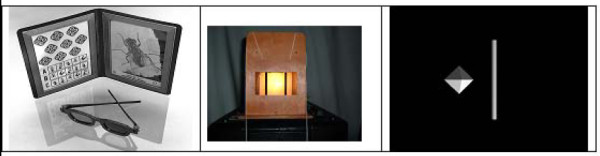
**Depth perception tests and examples of stimuli**. Titmus stereo test (left), Modified Howard-Dolman Apparatus (middle), Virtual Howard-Dolman stimuli (right).

Figure 4 displays each of the stereoacuity assessments performed during the experiment. Prior to testing, participants were screened using the Titmus Stereo Test, a standard projected-depth test, to confirm that their stereoacuity was at least 40 arc seconds. These tests are shown in Figure 4a and 4b, respectively. Further, the modified Howard-Dolman peg test using the Howard Dolman apparatus was performed before and after VE testing to monitor changes in visual performance over the course of the experiment.

The stimuli presented during the V-HD task are pictured in Figure 4c. The V-HD task controls for the familiar size cues by presenting generic objects, an octahedron and a cylinder), which have no real world correlation [32]. Thus, there is no expectation of size when simultaneously viewing both objects. However, aspects of the graphics such as lighting may provide a weak depth cue. Rolland et al [33] adjusted for conflicts between accommodation and convergence mechanisms of the human eye by placing the microdisplay with regard to the optics such that the monocular optical images matched the location at which the 3D virtual objects were rendered.

### Participants

This research was approved by the Institutional Review Board (IRB) of the University of Central Florida. Ten healthy male participants were randomly placed in either the HWPD-1 (mean age = 29.8, SD = 5.26) or HWPD-2 (mean age = 30.6, SD = 5.36) viewing group. The Titmus Stereo Test confirmed that participants' stereoacuity was at least 40 arc seconds prior to the start of the experiment. As well, each participant was either corrected for or had 20/20 vision. If needed, glasses or contacts were worn during each part of the experiment.

### Procedure

In this experiment, the participant performed the virtual Howard-Dolman (V-HD) task for two trials at a viewing distance of 800, 1500 or 3,000 mm. This viewing distance was randomly selected and each participant repeated the experiment on separate days until the stereoacuity assessment was performed at each distance. Before and after each virtual trial, the participant performed the modified Howard-Dolman task at the same viewing distance for that testing session to monitor possible changes in the participant's stereoacuity due to the VE exposure.

When performing the V-HD task, the HWD was adjusted for each person based upon comfort as well as IPD for each viewing distance. The virtual cylinder (target) was rendered at the chosen focus plane (800, 1500, or 3000 mm) and kept stationary. The virtual octahedron was randomly placed to the right or to the left of the cylinder, as well as in front of or behind the target object. The participant moved the octahedron using a dial so that its center was aligned with the center of the cylinder.

The response variables for this assessment were: 1) percent correct for whether the octahedron appeared in front of or behind the target; 2) the absolute constant error defined as the magnitude of the offset between the aligned objects; and, 3) the variable error or measure of dispersion about the participant's mean error score. The equivalence disparity metric (η), a measure of stereoacuity, was calculated from the absolute constant error and the variable error metrics [46,38]. The percent correct for front and back responses with the stereoacuity values are reported and discussed for each HWD tested.

## Results

### Stereoacuity calculated for HWPD-1 and HWPD-2

**Figure 5:**
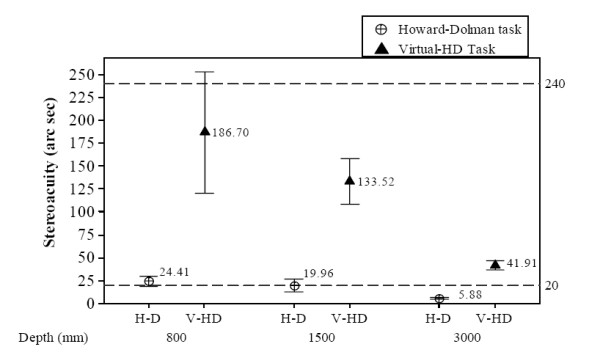
**HWPD-1 stereoacuity results**. HWPD-1-Overall stereoacuity (*η*) means and 95% Confidence Interval for the Howard-Dolman and Virtual-HD task at viewing distances of 800, 1500, 3000 mm.

**Figure 6:**
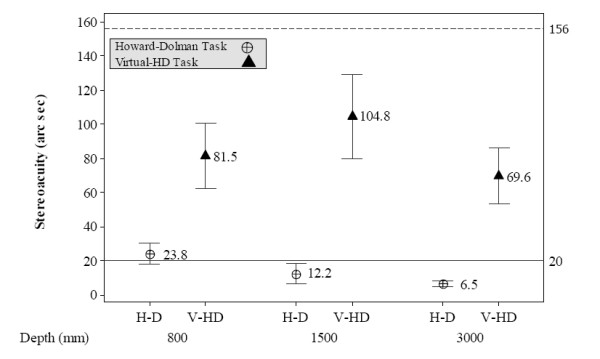
**HWPD-2 stereoacuity results**. HWPD-2 Overall stereoacuity (*η*) means and 95% Confidence Interval for the Howard-Dolman and Virtual-HD task at viewing distances of 800, 1500, 3000 mm.

Figure 5 and 6 show the overall mean stereoacuity values attained at each viewing distance, for each task, and each HWPD. The bottom reference line at 20 arc seconds represents typical stereoacuity values for the Howard-Dolman task. In Figure 5, the reference line at 240 arc seconds represents the predicted stereoacuity based on the size of a single pixel as determined by HWPD-1 parameters, which was previously given as 4.1 arc minutes. The predicted stereoacuity for HWPD-2 is 156 arc seconds or 2.73 arc minutes and appears as the reference line in Figure 6.

### Percent correct front and back for HWPD-1 and HWPD-2

**Figure 7:**
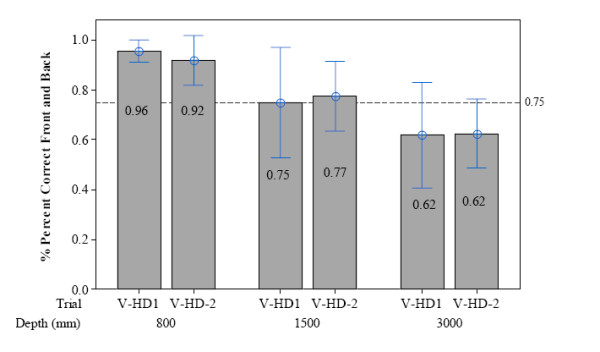
**HWPD-1 performance measures**. HWPD-1-Mean percent correct and 95% CI for front and back judgments on both trials of the V-HD task over each viewing distance.

**Figure 8:**
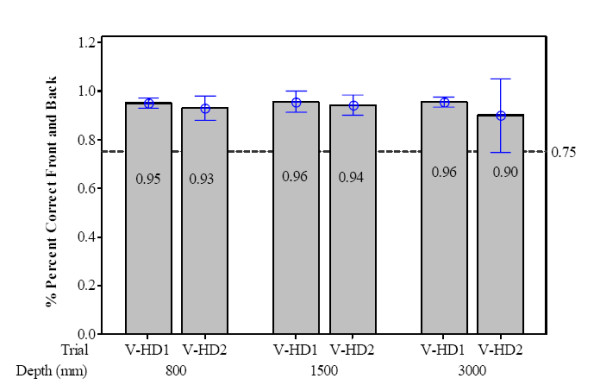
**HWPD-2 performance measures**. HWPD-2- Mean percent correct and 95% CI for front and back judgments on both trials of the V-HD task over each viewing distance.

The mean percent correct for responding whether the octahedron appeared in front of or behind the static cylinder prior to alignment is shown in Figure 7 and 8 for HWPD-1 and HWPD-2, respectively. This measure represents a 2 alternative-forced-choice (AFC) response where any score 75 percent and above meets the detection threshold. This threshold is indicated by dotted lines in both figures.

## Discussion

One aim of this study was to introduce a stereoacuity test capable of benchmarking HWDs. Stereoacuity of each HWD was evaluated given their respective display parameters utilizing a user-in-the-loop methodology. The results showed that there was no significant difference between groups when performing the Howard-Dolman task at any viewing distance. Thus, subsequent differences found between the groups may be attributed to the type of HWPD worn while performing the virtual Howard-Dolman task.

As figures 5 and 6 show, the participants' performance was better than the predicted stereoacuity based on the pixel size resolution of each display, 240 and 156 arc seconds, respectively. Participants wearing HWPD-1 performed more variably at the 800 mm viewing distance; however, as the distance was adjusted toward the optimized optical plane, participants' performance improved significantly, (M_V-HD800 _= 186.70 arc sec, SD = 92.10 arc sec; M_V-HD1500 _= 133.52 arc sec, SD = 34.6; M_V-HD3000 _= 41.91 arc sec, SD = 7.18). This result is expected since the HWPD-1 is designed to perform optimally at infinity viewing conditions. In contrast, stereoacuity for persons wearing HWPD-2, which is optically optimized across each viewing distance, does not change significantly with viewing distance, M_V-HD800 _= 81.46, SD = 27.01, M_V-HD1500 _= 104.80, SD = 34.70, M_V-HD3000 _= 69.6, SD = 23.05.

These results suggest that HWPD-1 may not be the best candidate HWD for performing tasks requiring good stereoacuity in personal space (i.e., within arms reach). However, HWPD-1 does attain stereoacuity levels closer to those attained under natural viewing conditions when the optical depth plane is set to infinity or the setting for which it was optimized. The stereoacuity levels for HWPD-2 are not maximized for any one viewing distance. It is known that stereoacuity improves with improved binocular visual acuity [47,48]. Thus, although HWPD-2 provides better binocular visual acuity than HWPD-1, this advantage is diminished for the 3000 mm condition because of the requirement of optimizing the optics across the additional optical plane settings. This finding points to the benefit of designing HWDs to target a specific field of use for which visual task performance must be optimized.

It should also be noted that the stereoacuity scores obtained when wearing either HWPD are lower than the predicted real-world stereoacuity values for the same levels of visual acuity attainable by each HWPD. Real world stereoacuity predictions for a Snellen score of 20/80 range from 178 to 200 arc seconds, which matches the visual acuity attainable by HWPD-1. For a Snellen score of 20/60, which corresponds to HWPD-2, predicted stereoacuity values range from 160 to 200 arc seconds [47,49]. Figures 5 and 6 show that HWPD-1 and HWPD-2 match or best these predicted values. This improvement in stereoacuity is attributed to antialiasing techniques which improve the visibility of edges of the rendered objects. This result suggests interdependence between image resolution of the rendered virtual objects and computer graphics techniques that should be accounted for when assessing VE systems for rehabilitation therapies.

Figures 7 and 8 display the percent correct responses for determining whether the octahedron appeared in front of or behind the cylinder before aligning the objects while performing the task wearing HWPD-1 or HWPD-2. While wearing HWPD-1, the participants were able to perform above threshold for the 800 and the 1500 mm viewing distances; however, they failed to meet threshold for the 3000 mm viewing distance. As Figure 7 shows, participants performed similarly for both repetitions of the task at the 3000 mm viewing distance, M_V-HD1 _= .62, SD = .17 and M_V-HD2 _= .62, SD = .11. Thus, the task was difficult for participants to perform even with prior exposure to the test stimuli at that distance.

In comparison, Figure 8 shows that participants did not respond below 90% correct when evaluating front and back initial positions of the octahedron relative to the fixed cylinder while performing the task wearing HWPD-2. There are several properties of HWPD-2 that may contribute to better performance. First, the display type is an OLED, Organic Light Emitting Diode, which provides more brightness and contrast than the standard LCD, Liquid Crystal Display, of HWPD-1 (Cakmakci, 2006). As well, the increased resolution acuity of the HWPD-2 also lends to an improved resolvable depth (i.e., the range of distances in front of and behind the optical plane where a single pixel size is just resolvable) over HWPD-1.

These results suggest that HWD characteristics reduce stereoacuity for persons with unimpaired vision. This reduction is quantifiable using the virtual Howard-Dolman task. Rehabilitation specialists can compare these benchmarks to assess the appropriateness of different HWDs for use in their VR protocols. For example, HWPD-1 may not be an appropriate choice for close distance therapeutic tasks (e.g., dropping a letter in a virtual mailbox) as the stereoacuity limitations may augment the patient's propensity for errors. These design decisions can occur early in the design process, thus reducing time and cost while potentially improving the overall effectiveness of the VR therapy.

## Conclusions

HCI guidelines are important in VE systems design; however, their goals and methods may change to accommodate for the sensory and perceptual deficits of persons with cognitive impairment. For example, persons with anterograde amnesia may not remember their VE exposure even 10 minutes post training. Questionnaires or verbal report methods may not be appropriate for this population; however, procedural tasks that support motor memory may prove useful. Thus, VE design for VR must match research concerns with the comfort, the safety, and the acceptability of the user. Research and safety constraints may be best satisfied with baseline metrics representing both clinical and unimpaired populations.

Task characteristics also determine the level of realism, immersion, and overall detail necessary to achieve the desired training effect. For example, pain management and exposure therapies using VE technologies do not demand high levels of graphical detail or realism to achieve positive behavioral outcomes [50,51]. However, rehabilitation protocols for assisting persons with spatial neglect should ensure that distortions of shape and size constancy, as well as depth errors caused by the VE technology are controlled or at least understood prior to therapy and experimentation.

As more technology providers (e.g., haptic devices) follow, convergence between the engineering and user requirements will simplify the VE design decision making. However, benchmarks addressing user sensory capabilities across separated and integrated technologies are necessary when evaluating user performance, especially when evaluating persons with brain injuries. These benchmarks are critical to evaluating negative adaptation effects as well as positive transfer of training.

More specifically, we argue that current user-centered design approaches are too high level to support the empirical nature of VR. When building a specific rehabilitation application, it is appropriate to introduce sensory performance assessments after the initial task analysis is performed. However, general sensory baselines attained via a user-in-the-loop methodology are the responsibility of the device manufacturer. The goal of the therapist is not to determine the individual limits of the technology from a user perspective, but to define the integrative effects of technology as they assist or detract from user performance in a rehabilitation scenario.

## Competing interests

The authors declare that they have no competing interests.

## Authors' contributions

AAR provided the foundational concepts for VR system design, including benchmarking. CMF conceived of the additions to the design cycle, participated in the design of the stereoacuity test, implemented the study, performed the analyses, and drafted the manuscript. JPR conceived of the study along with the stereoacuity test and the experimental design. JPR also assisted with data analyses and the editing of the manuscript. All authors read and approved the final manuscript.
